# Multigenerational variation in the nutrients and digestion of western flower thrips (*Frankliniella occidentalis*) depends on the nutritive quality of different foods

**DOI:** 10.1093/jisesa/iead020

**Published:** 2023-04-13

**Authors:** Wen Bo Yue, Dan Zhou, Ding Yin Li, Jun Rui Zhi, Xin Lei Fang, Xin Yue Qiu

**Affiliations:** Institute of Entomology, Guizhou University, Guizhou Key Laboratory for Agricultural Pest Management of the Mountainous Region, Guiyang 550025, China; Institute of Entomology, Guizhou University, Guizhou Key Laboratory for Agricultural Pest Management of the Mountainous Region, Guiyang 550025, China; Qingzhen City Agricultural and Rural Bureau, Guiyang, Guizhou 551400, China; Institute of Entomology, Guizhou University, Guizhou Key Laboratory for Agricultural Pest Management of the Mountainous Region, Guiyang 550025, China; Institute of Entomology, Guizhou University, Guizhou Key Laboratory for Agricultural Pest Management of the Mountainous Region, Guiyang 550025, China; Institute of Entomology, Guizhou University, Guizhou Key Laboratory for Agricultural Pest Management of the Mountainous Region, Guiyang 550025, China; Institute of Entomology, Guizhou University, Guizhou Key Laboratory for Agricultural Pest Management of the Mountainous Region, Guiyang 550025, China

**Keywords:** thrips, adaptation, kidney bean pods, rose flowers, nutrient

## Abstract

Western flower thrips (WFTs), *Frankliniella occidentalis* (Thysanoptera, Thripidae), is one of the most serious pests that attack rose flowers. Little is known about the effect of different parts of the rose flower on nutritional contents and digestive enzyme activities in thrips. This study assessed variations in the nutritional contents and digestive enzyme activities in the second-instar larvae and adults WFTs fed on 3 food types (rose petals, rose flowers, and honey solution + kidney bean pods) for multiple generations. The highest contents of soluble sugar (in 10% honey solution + kidney bean pods), amino acid (in rose flowers), and protein (in rose flowers) were observed, respectively. Soluble sugar and protein contents in the second-instar larvae and adults fed on rose petals decreased in the F_1_ generation but increased in the F_2_ generation and remained at higher levels until the F_7_ generation. Feeding of thrips with 3 food types increased the lipid content in the F_1_ generation, which peaked in the F_2_ generation and remained high until the F_7_ generation. In most cases, α-amylase and trypsin activities significantly decreased in the F_1_ generation after feeding on rose petals and then prominently increased in the F_2_ generation. In contrast, chymotrypsin activity remarkably increased and peaked in the F_1_ generation after second-instar larvae thrips fed on rose petals. There were correlations among the contents of 3 nutrient related positively with the activities of α-amylase and trypsin in WFTs second-instar larvae and adults, respectively. Overall, variations in the nutrient properties of the 3 food types caused changes in nutrient contents and digestive enzyme activities in thrips.

## Introduction

Insects are one of the largest and most diverse group of organisms in nature, and their varied feeding characteristics have contributed to the insect–plant coevolution process. They obtain energy through feeding on multispecial plant foods and preys for their growth and development (Coll and Guershon 2002, Gutierrez et al. 2020). The history of regular food stresses and unpredictability has shaped the diverse insect feeding mechanisms. To overcome and adapt to the challenges of food nutrient variation caused by environmental changes, insects have undergone complex feeding, developmental, and reproductive processes as well as physiological and biochemical changes to ensure species survival (Vankosky and VanLaerhoven 2015, Li et al. 2017, Gutierrez et al. 2020).

Different food types contain diverse nutrient components (Wu et al. 2007, Cao et al. 2011). This can directly affect the content of energy substances in insects (Guo et al. 2018, He et al. 2019), which in turn affects the growth, development, weight, and reproduction of insects (Lin et al. 2003, Cao et al. 2015, Qin et al. 2017). The nutritional value of food is an important factor that can determine the successful life-cycle completion and reproduction of insects. Sugars, proteins, and lipids are basic nutrients and crucial sources of energy obtained by insects from host plants or other food types for normal growth, development, and reproduction (Qin and Wang 2001, Hódar et al. 2002). Soluble sugar is also an important source of nutrients and energy, and its presence in food can stimulate insect feeding and infestation (Gonzalez et al. 1989, Oroño et al. 2018). Higher contents of soluble protein in food relatively improve the survival rate, growth, and development as well as fecundity of insects (Mattson 1980, Awmack and Leather 2002). Amino acids are one kind of the essential nutrients for insect growth and development. Insects regulate the expression of nonessential amino acids based on their needs and utilization of amino acids in food, subsequently affecting various metabolic pathways and resulting in spawning differences (Subramanyam et al. 2018). Variation in food nutrient contents can lead to differences in insect nutrient content, resulting in modified growth, development, and reproduction of insects (Cao et al. 2015, Qin et al. 2017). Nutritional indexes can reflect the host plant’s suitability for insects (Zhang et al. 2018).

Digestive enzymes are important physiological components that play pivotal roles in the maintenance of normal growth, development, and survival of insects as well as their physiological and biochemical metabolism (Terra and Ferreira 1994, Erthal et al. 2007, Zamani Fard et al. 2022). Therefore, digestive enzyme activity is often used as a key indicator for determining the physiological adaptability of insects (Yuan et al. 2020, Zhi et al. 2021, Hemmati et al. 2022). The predominant digestive enzymes in herbivorous insects include proteases and amylases, whereas those in carnivorous insects mainly include proteases and lipases; moreover, trehalases, and invertases are other important enzymes in insects (Qin and Wang 2001, Li 2015). The digestive enzymes in insects act synergistically to digest and metabolize sugars, cellulose, proteins, and lipids ingested from host plants or other foods to provide energy for normal life activities.

Western flower thrips (WFTs), *Frankliniella occidentalis*, also known as alfalfa thrips, belong to the order Thysanoptera in the family Thripidae. *F. occidentalis* is a major insect pest of worldwide economic significance (Mouden et al. 2017, Reitz et al. 2020). WFTs have a wide range of hosts, including vegetables, fruits, flowers, and other crops (Funderburk 2009, Reitz 2009, Reitz et al. 2020). They have diverse diets and feed on plant sap as well as pollen, nectar, and eggs of mites (Kirk 1985, Trichilo and Leigh 1986, Zhi et al. 2005). The type of host plants, different parts of host plants, and presence or absence of flowers affect the ecological, physiological, and biochemical characteristics of thrips (Agrawal et al. 1999, Gerin et al. 1999, Mouden et al. 2017). Some studies have reported the responses of digestive enzymes of WFTs to feeding on different vegetables and flowers (Zhi et al. 2021, Qi et al. 2022), and their population burst has closely been associated with the blooming of flowers (Sun et al. 2011). Therefore, we hypothesized that the nutrient contents of rose flowers, pollen, and nectar affect the nutrient contents of WFTs and their digestive enzyme activities, which could contribute to their feeding choice, physiological metabolic mechanism, and population dynamics.

Limited information is currently available regarding the effects of different flower parts on nutritional content and digestive enzyme activities in WFTs. This study used petals and flowers (including pollen and nectar) of the *Rosa rugosa* plant as well as 10% honey solution + kidney bean pods as food types to evaluate the changes in nutrients and digestive enzyme activities of thrips in different generations. The knowledge of the relationship between food nutrient content and digestive enzymes of WFTs can enhance the understanding of their physiological adaptive mechanisms to diverse food types. Furthermore, this study provides a theoretical reference for clarifying the outbreak patterns of WFTs for their comprehensive management.

## Materials and Methods

### Thrips Colonies

The western flower thrips (WFTs), *Frankkliniella occidentalis*, were collected from bean plants in Huaxi District, Guiyang City, Guizhou Province and reared with kidney bean (*Phaseolus vulgaris*) pods for over 30 generations in an artificial climate box (RXZ type, Ningbo Jiangnan Instrument Factory, China). The feeding conditions were as follows: temperature, 25 °C ± 1 °C; humidity, 70% ± 5%; and photoperiod, light: dark cycle of 14:10 h.

### Different Food Types

#### Kidney bean pods

 Pods of stemless kidney bean varieties were purchased from local farmers in Huaxi District, Guiyang City, Guizhou Province, China. The kidney bean pods were repeatedly washed with running water; they were then dried before use.

#### Honey

Rape flower honey (Jieshi Co., Ltd.) was purchased from the supermarket, and 100 ml of honey was added to 900 ml of distilled water to obtain 10% (v/v) honey solution.

#### 10% honey solution + bean pods

Clear kidney bean pods were soaked in 10% honey solution for 10 min; they were taken out of the solution and dried before use.

#### Rose plants

The *Rosa rugosa* of the dark red variety was purchased from Yunnan Xinhaihui Flower Industry Base, China, and then planted in a disease and insect pest free greenhouse, and no pesticides were sprayed during the growth period.

#### Rose petals

Only rose petals were selected as test food, and the stamens and pistils were removed.

#### Rose flowers

The whole rose flower, including petals, stamens, pistils, pollen, and nectar, was used, and the proportion of petals and pollen nectar was 5:1 (w/w).

### Samples of the 4 Food Types

Fresh bean pods, rose petals, and rose flowers were cut into pieces, and 0.1 g of each food type was used for determining soluble sugar contents. Similarly, 0.1 g of each food type was used for determining amino acid contents, whereas 0.05 g of each food type was used to determine protein contents. All samples were prepared in 3 biological replicates, transferred to 2-ml centrifuge tubes, and stored at −80 °C for subsequent use.

### Determination of Nutrient Content in Different Food Types

Soluble sugar content was determined using the anthrone–sulfuric acid colorimetric assay (Tang et al. 2021, Zhang et al. 2021). Under the action of concentrated sulfuric acid, sugar is dehydrated to form furfural or hydroxymethyl furfural, which reacts with anthrone to form a blue–green substance; the absorbance peak is observed at a wavelength of 620 nm. Soluble sugar content is then calculated based on the absorbance value.

Amino acid content was determined via a colorimetric assay (Moore et al. 1951, Guo et al. 2018). In this assay, the α-amino group of amino acids reacts with hydrated ninhydrin to form blue-purple compounds, which show a distinctive absorption peak at 570 nm. This value can be used to calculate the content of amino acids.

Protein content was determined using the Coomassie brilliant blue G-250 assay (Bradford 1976, Cao et al. 2011). Coomassie brilliant blue G-250 can bind to proteins to form a blue complex, with a maximum absorption peak at 620 nm, which can be used to calculate the protein content.

The abovementioned assays were performed using commercial kits (Suzhou Comin Biotechnology, Suzhou, China), according to the manufacturer’s instructions.

### Treatment of WFTs

Several male and female adults were fed on kidney bean pods for over 30 generations, emerged for 3 days and mated, and then placed in boxes (length, 20 cm; width, 13 cm; height, 9 cm) containing 3 different food types (rose petals, rose flowers, and 10% honey solution + kidney bean pods), respectively. Fresh foods were replaced every 24 h, and those with eggs within plant tissue were placed into a new clean box for hatching. The hatched eggs were allowed to develop into second-instar larvae, which were considered as F_1_ larvae; furthermore, after developing into adults, they were termed as F_1_ adults. The F_1_ adults were fed with the same food types to allow their egg to hatch, resulting in F_2_ second-instar larvae, which developed into F_2_ adults. This cycle was repeated until the F_7_ adults were obtained. The 50 second-instar larvae or 50 adults were collected in the F_1_, F_2_, F_3_, F_5_, and F_7_ generations fed on 3 food types, respectively. Feeding thrips with kidney bean pods was used as the control group, 50 second-instar larvae and 50 adults were collected. Each of treatment was provided 3 replicates.

### Sample Collection

Overall, 50 second-instar larvae and 50 adults WFTs fed on different foods in each generation were selected, placed in 2-ml centrifuge tubes, and stored at –80 °C for subsequent determination of various digestive enzyme activities in 3 replicates. In addition, 100 thrips of each treatment were used for determining various nutrient contents, respectively.

### Determination of Digestive Enzyme Activity in Thrips

#### Preparation of digestive enzyme solution

For determining α-amylase activity, 500 μl of distilled water was added to a centrifuge tube containing 50 WFTs. Samples were homogenized using a high throughput tissue grinder, and the homogenate was transferred to a 1.5-ml centrifuge tube and was extracted at room temperature for 15 min. The enzyme solution was then centrifuged for 10 min at 8,000 rpm and 25 °C, and the supernatant was used as the enzyme solution. For determining trypsin and chymotrypsin activities, 500 μl of precooled physiological saline (0.4%) was added to a centrifuge tube containing 50 WFTs. Samples were homogenized in an ice bath using a high-flux tissue grinder, and the solution was centrifuged for 10 min at 10,000 rpm and 4 °C. Then, the supernatant was used as the enzyme solution for subsequent analyses.

#### Determination of the enzyme activity

All digestive enzyme activity assays were conducted using commercial kits (Suzhou Keming Biotechnology Co., Ltd), according to the manufacturer’s instructions.

##### α-Amylase activity

The determination of α-amylase activity was based on the principle of reduction of 3,5-dinitrosalicylic acid. α-amylase can catalyze the hydrolysis of starch to produce reducing sugar. The reduced sugar further reduces 3,5-dinitrosalicylic acid to produce brownish red substances, which show peak absorption at 540 nm, and the α-amylase activity can be calculated by measuring the change in absorbance.

##### Trypsin activity

The enzyme activity was determined using the principle of catalytic hydrolysis of the ester bond of p-toluenesulfonyl-l-arginine methyl ester solution (TAME) (Wang and Qin 1996). Trypsin catalyzes the hydrolysis of the ester bond of TAME, and the released carboxyl group neutralizes with sodium hydroxide, resulting in decreased pH of the solution. Phenol red is used as an indicator to calculate the trypsin activity based on the change in absorption at 555 nm.

##### Chymotrypsin activity

The enzyme activity was determined using the principle of catalytic hydrolysis of N-benzoyl-l-tyrosine ethyl ester (BTEE). The hydrolysate of BTEE shows a characteristic light absorption at 237 nm. The increasing rate of light absorption at 237 nm is measured and used to estimate the chymotrypsin activity.

##### Protein content

The Coomassie brilliant blue G-250 assay was used to determine the protein content in insects (Bradford 1976). In total, 100 WFTs were placed in a centrifuge tube containing 1 ml of distilled water and were then homogenized in an ice bath using a high-flux tissue grinder. The homogenate solution was centrifuged for 10 min at 8,000 rpm and 4 °C, and the liquid supernatant was collected for protein content determination, according to the manufacturer’s instructions (Suzhou Keming Biotechnology Co., Ltd.). The results were expressed as μg/thrips.

### Determination of Nutrient Contents in Thrips

#### The contents of soluble sugar and lipid

For sample preparation, 50 μl of 2% Na_2_SO_4_ solution was transferred to a centrifuge tube containing 100 WFTs in an ice bath, and the homogenate was grounded. Next, 450 μl of chloroform: methanol (volume ratio of 1:2) solution was added to the homogenate, which was centrifuged for 15 min at 12,000 rpm. The supernatant was subsequently collected for the measurement of soluble sugar and lipid contents in insects.

##### Soluble sugar content

The anthrone–sulfate assay (Van Handel 1985a) was used to estimate soluble sugar content in thrips. Briefly, 100 μl of the test solution was added to a 2-ml centrifuge tube in an ice bath, and 1 ml of anthrone was added and thoroughly mixed. The solution was subsequently transferred to a water bath at 90 °C for 10 min and then cooled to room temperature. Approximately 200 µl of the reaction solution was added to the enzyme plate, and the absorption value was obtained at 630 nm. The standard curve was constructed using glucose standards. The results are expressed as g/thrips.

##### Lipid content

The sulfo-phospho-vanillin reaction (Van Handel 1985b) was used for analyzing the lipid content. Lipid was precipitated by adding 100 μl of concentrated sulfuric acid to a 2-ml centrifuge tube containing the sample. The solution was transferred to a water bath at 90 °C for 10 min and then cooled for 5 min to room temperature. Next, the solution was mixed with 1 ml of vanillin reagent, and 200 µl of the reaction solution was added to the enzyme standard plate after 15 min. The absorption value was recorded at 525 nm, and the standard curve was constructed using cholesterol standards. The results are expressed as g/thrips.

### Data Processing and Analysis

The data were statistically analyzed using Microsoft Excel 2016 and SPSS 26.0. Tukey’s test (*P* < 0.05) was used to analyze significant differences among different foods as well as those among nutrient contents and digestive enzyme activities in WFTs at *P* < 0.05. To determine the effects of feeding food types and generations on nutrient content and digestive enzyme activity in WFTs, two-way ANOVA was carried out. Pearson correlation was used to determine associations between nutritional content and digestive enzyme activity in WFTs. Graphs were obtained using GraphPad prism 9.4.

## Results

### Two-way ANOVA of 3 Nutrient Contents in *Frankliniella occidentalis* Based on Food Types and Generations

As shown in Table 1, the contents of 3 nutrient (soluble sugar, protein, and lipid) were significantly influenced by food types and generations in second-instar larvae, but the interaction between food types and generations did not significantly influence their soluble sugar and lipid contents. Similarly, the levels of 3 nutrients in adults were significantly influenced by food types and generations. But only the interaction between food types and generations did not significantly influence the contents of soluble sugar in adults.

**Table 1.** Two-way ANOVA of nutrient contents in *F. occidentalis* based on food types and generations

Source of variation	df	Second-instar larvae		Adults	
		*F*	*P*	*F*	*P*
Soluble sugar					
Food types	2	1134.515	**<0.001**	1587.551	**<0.001**
Generations	4	156.162	**<0.001**	228.373	**<0.001**
Food types × generations	8	1.172	0.348	2.005	0.800
Protein					
Food types	2	203.603	**<0.001**	191.868	**<0.001**
Generations	4	48.805	**<0.001**	39.944	**<0.001**
Food types × generations	8	4.955	**0.001**	2.458	**0.035**
Lipid					
Food types	2	138.215	**<0.001**	326.441	**<0.001**
Generations	4	32.970	**<0.001**	54.256	**<0.001**
Food types × generations	8	1.229	0.316	2.817	**0.019**

Significant effects (*P* < 0.05) are indicated in bold. Summary results following generalized linear model ANOVA in SPSS Version 26.

### Variation in Soluble Sugar Content Among Different Food Types

**Fig. 1.**
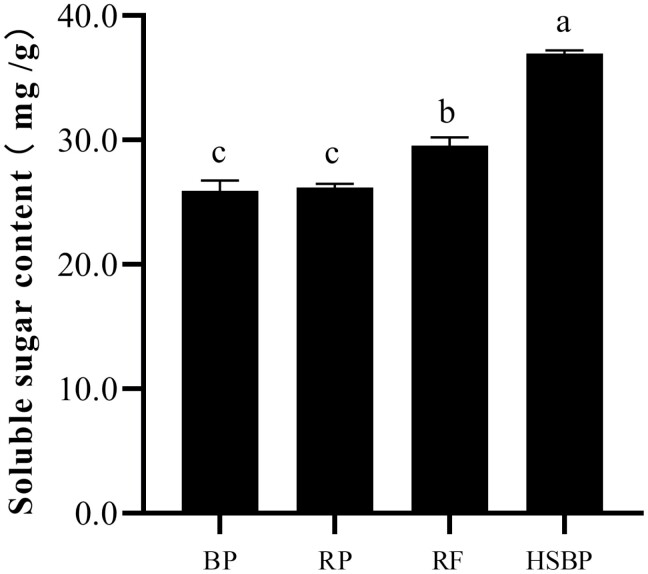
The content of soluble sugar in different food types. Different lowercase letters indicate significant differences among different food types at the *P* < 0.05 level by Tukey’s test. The error bars indicate the standard error of 3 replicates. The same as below.

Significant differences were observed in the soluble sugar contents of bean pods, rose petals, rose flowers (including pollen and nectar), and 10% honey solution + bean pods (Fig. 1). The highest soluble sugar content was observed in 10% honey solution + bean pods (36.94 mg/g), followed by 29.54 mg/g in rose flowers. Whereas the lowest soluble sugar contents were observed in bean pods (25.93 mg/g), and with no significant difference between bean pods and rose petals.

### Protein and Amino Acid Content in Different Food Types

**Fig. 2.**
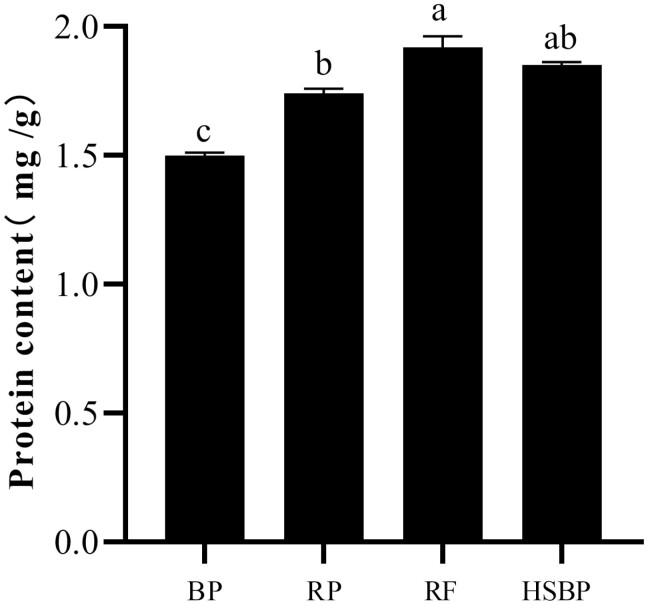
The content of protein in different food types.

**Fig. 3.**
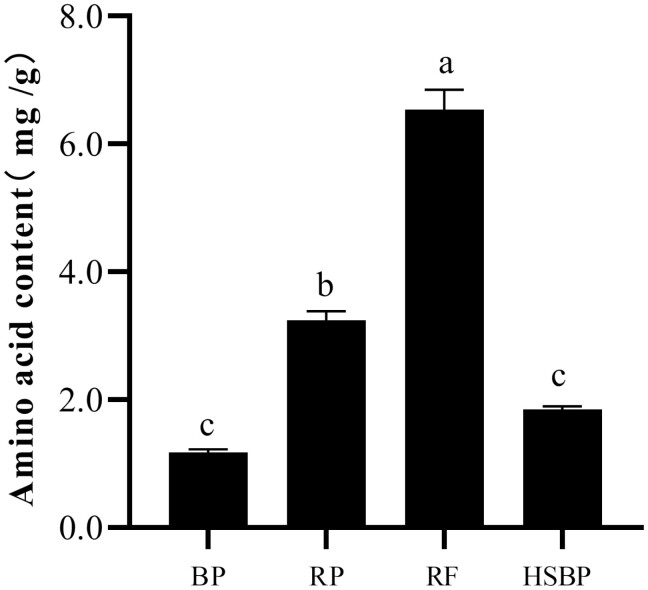
The content of amino acid in different food types.

Significant differences in protein and amino acid contents were observed in the 4 food types (Figs. 2 and 3). The highest protein contents were detected in rose flowers (1.92 mg/g) and 10% honey solution + kidney bean pods (1.85 mg/g), respectively. The protein content in rose petals was 1.74 mg/g, and no significant difference was observed between 10% honey solution + kidney bean pods and rose petals. In contrast, the lowest protein content was observed in kidney bean pods (1.50 mg/g).

Similarly, the highest amino acid content was detected in rose flowers (6.54 mg/g), followed by 3.24 mg/g in rose petals. In contrast, the lowest amino acid contents were detected in bean pods (1.18 mg/g) and 10% honey solution + bean pods (1.85 mg/g), respectively, with no significant difference between them.

### Effects of Food Types on the Soluble Sugar Content of WFTs in Different Generations

The soluble sugar contents of WFTs were different after being reared on the same food for multiple generations (Table 2). When fed on rose petals, soluble sugar content in the F_1_ generation was significantly lower than that in the control. However, soluble sugar content showed a significant increase in the F_2_ generation compared with the control, and this higher content was maintained in the F_3_, F_5_, and F_7_ generations. Similar changes in soluble sugar content were detected in second-instar larvae fed on rose flowers and 10% honey solution + bean pods. The soluble sugar content in the F_1_ generation was significantly higher than that in the control, and it continuously increased from the F_2_ generation until the F_7_ generation, with no significant differences among the 4 generations (F_2_, F_3_, F_5_, F_7_).

**Table 2.** Response of soluble sugar content in *F. occidentalis* feeding on different foods for different generations

Food types	CK (kidney bean pods)	Soluble sugar (µg/thrips)				
		F_1_	F_2_	F_3_	F_5_	F_7_
Second instar larvae						
Rose petals	2.53 ± 0.05b	2.14 ± 0.11cC	3.13 ± 0.08aC	3.28 ± 0.08aC	3.32 ± 0.07aC	3.33 ± 0.04aC
Rose flowers	2.53 ± 0.05c	3.51 ± 0.05bB	4.49 ± 0.04aB	4.49 ± 0.06aB	4.56 ± 0.06aB	4.51 ± 0.08aB
10% Honey solution + kidney bean pods	2.53 ± 0.05c	4.16 ± 0.04bA	5.06 ± 0.06aA	5.22 ± 0.07aA	5.06 ± 0.05aA	5.23 ± 0.03aA
Adults						
Rose petals	3.92 ± 0.09b	3.26 ± 0.06cC	4.43 ± 0.06aC	4.45 ± 0.03aC	4.44 ± 0.03aC	4.40 ± 0.06aC
Rose flowers	3.92 ± 0.09c	4.72 ± 0.09bB	6.28 ± 0.06aB	6.36 ± 0.03aB	6.33 ± 0.07aB	6.26 ± 0.06aB
10% Honey solution + kidney bean pods	3.92 ± 0.09c	5.52 ± 0.09bA	7.07 ± 0.09aA	7.06 ± 0.13aA	7.09 ± 0.11aA	6.97 ± 0.04aA

Data in the table are mean ± SE. Different lowercase letters indicate significant differences in the same row, and different uppercase letters indicate significant differences in the same column in second-instar larvae or adults. The Tukey’s test was used to analyze significant differences at the *P* < 0.05 level.

Soluble sugar contents in second-instar larvae were different between the 3 food types within the same generation, although changes in its contents in second-instar larvae were similar in all generations. Soluble sugar contents increased with feeding in the order of rose petals < rose flowesr < 10% honey solution + bean pods, with rose petals feeding exhibiting the lowest content. Changes in soluble sugar content in adults thrips fed with various foods in different generations were similar to those in second-instar larvae.

### Effects of Food Types on Protein Content of WFTs in Different Generations

The protein content in second-instar larvae fed on rose petals in the F_1_ generation was lower than that in the control (Table 3). In contrast, the protein content in second-instar larvae after feeding in the F_2_ generation was significantly higher than that in the control, with no significant difference among the 4 generations (F_2_, F_3_, F_5_, and F_7_). The protein content in second-instar larvae fed on rose flowers and 10% honey solution + bean pods in the F_1_ generation was significantly higher than that in the control. The higher protein content obtained in the F_2_ generation remained stable thereafter.

**Table 3.** Responses of protein content in *F. occidentalis* feeding on different foods for different generations

Food types	CK (kidney bean pods)	Protein (µg/thrips)				
		F_1_	F_2_	F_3_	F_5_	F_7_
Second instar larvae						
Rose petals	9.62 ± 0.09b	8.29 ± 0.27cC	11.23 ± 0.21aB	11.39 ± 0.32aB	11.24 ± 32aB	11.07 ± 0.26aB
Rose flowers	9.62 ± 0.09c	12.54 ± 0.15bA	13.32 ± 0.20aA	13.37 ± 0.25aA	13.53 ± 0.08aA	13.41 ± 0.11aA
10% Honey solution + kidney bean pods	9.62 ± 0.09c	11.20 ± 0.23bB	13.28 ± 0.16aA	13.48 ± 0.26aA	13.11 ± 0.23aA	13.51 ± 0.10aA
Adults						
Rose petals	12.43 ± 0.06b	11.43 ± 0.18cB	12.56 ± 0.07bC	13.48 ± 0.26aC	13.54 ± 0.10aC	13.40 ± 0.19aC
Rose flowers	12.43 ± 0.06c	13.87 ± 0.27bA	15.60 ± 0.11aA	15.71 ± 0.07aA	15.86 ± 0.19aA	15.54 ± 0.27aA
10% Honey solution + kidney bean pods	12.43 ± 0.06b	13.28 ± 0.05bA	14.45 ± 0.23aB	14.43 ± 0.26aB	14.30 ± 0.09aB	14.23 ± 0.31aB

Data in the table are mean ± SE. Different lowercase letters indicate significant differences in the same row, and different uppercase letters indicate significant differences in the same column in second-instar larvae or adults. The Tukey’s test was used to analyze significant differences at the *P* < 0.05 level.

After feeding on the 3 food types, the protein content in the F_1_ generation second-instar larvae increased in the order of rose flowers > 10% honey solution + bean pods > rose petals. After F_2_ generation, the protein content in thrips fed on the 3 food types was similar, whereas that in second-instar larvae was significantly lower after feeding on rose petals. In addition, feeding of thrips with rose flowers and 10% honey solution + bean pods led to an increase in their protein contents, with no significant difference between the 2 food types.

Protein content in adults fed on rose petals decreased in the F_1_ generation and increased to levels similar to that in the control in the F_2_ generation. Higher protein contents were obtained in the F_3_ generation, which remained similar in the F_5_ and F_7_ generations. When thrips were fed on rose flowers, the protein content in adults steadily increased from the F_1_ to F_2_ generations; however, no differences in protein content were observed among adults of the F_2_, F_3_, F_5_, and F_7_ generations. Feeding of adults on 10% honey solution + bean pods resulted in similar protein content in the F_1_ generation and control; however, in the F_2_ generation, the protein content in adults was significantly higher than the control. After the F_2_ generation, no significant changes in protein contents were observed among the adults of subsequent generations.

The lowest protein content was observed in F_1_ adults thrips fed on rose petals, whereas the protein content in thrips fed on other 2 food types was not significantly different. After feeding for F_2_–F_7_ generations with 3 food types, changes in protein content among adults thrips were similar, which showed an increase in the order of feeding rose flowers > 10% honey solution + bean pods > rose petals.

### Effects of Food Types on Lipid Content of WFTs in Different Generations

The changes in lipid content in second-instar larvae fed on rose petals and 10% honey solution + bean pods were similar with different generations (Table 4), and the lipid content was significantly lower in the F_1_ generation. When thrips were fed on 2 food types, the lipid content in F_1_ second-instar larvae significantly decreased compared with that in the control; however, it increased in the F_2_ generation, with no significant difference in the contents among the subsequent generations. Before F_3_ generation, the increase in lipid content in second-instar larvae after feeding on rose flowers was similar to that after feeding on other 2 food types. However, the lipid content in second-instar larvae decreased in F_5_ and F_7_ generations but remained higher than that in the control.

**Table 4.** Responses of lipid content in *F. occidentalis* feeding on different foods for different generations

Food types	CK (kidney bean pods)	Lipid (µg/thrips)				
		F_1_	F_2_	F_3_	F_5_	F_7_
Second instar larvae						
Rose petals	8.04 ± 0.30c	9.30 ± 0.18bC	11.55 ± 0.19aB	11.20 ± 0.28aB	11.15 ± 0.23aB	11.52 ± 0.23aB
Rose flowers	8.64 ± 0.30c	12.16 ± 0.23bA	13.72 ± 0.26aA	13.50 ± 0.28aA	13.40 ± 0.27abA	13.35 ± 0.24abA
10% Honey solution + kidney bean pods	8.64 ± 0.08c	11.02 ± 0.21bB	12.37 ± 0.13aB	12.16 ± 0.11aB	12.54 ± 0.18aA	12.63 ± 0.12aA
Adults						
Rose petals	10.41 ± 0.26c	11.81 ± 0.18bC	13.54 ± 0.14aC	13.03 ± 0.17aC	13.44 ± 0.12aC	13.63 ± 0.13aC
Rose flowers	10.41 ± 0.26c	14.49 ± 0.16bA	15.64 ± 0.15aA	16.27 ± 0.19aA	16.17 ± 0.11aA	15.90 ± 0.18aA
10% Honey solution + kidney bean pods	10.41 ± 0.26d	13.50 ± 0.16bB	14.33 ± 0.15baB	14.96 ± 0.15aB	15.04 ± 0.20aB	15.17 ± 0.20aB

Data in the table are mean ± SE. Different lowercase letters indicate significant differences in the same row, and different uppercase letters indicate significant differences in the same column in second-instar larvae or adults. The Tukey’s test was used to analyze significant differences at the *P* < 0.05 level.

Comparison of the lipid content in thrips among the 3 food types showed that it varied in different generations. In the F_1_ generation, lipid content in second-instar larvae increased with feeding in the order of rose flowers >10% honey solution + bean pods > rose petals. In the F_2_ and F_3_ generations, the lipid content in second-instar larvae fed on rose flowers was significantly higher than that in those fed on other 2 food types. In contrast, in the F_5_ and F_7_ generations, the lipid content in second-instar larvae fed on rose petals was significantly lower than that in those fed on other 2 food types.

Changes in lipid content in adult thrips fed on the same food were similar to those in second-instar larvae in different generations. After feeding on the 3 food types, an obvious increase in lipid contents was observed in the F_1_ generation, which remained stable after the F_2_ generation. Changes in lipid contents in adult thrips fed on the 3 food types in all generations were similar, and the lipid content increased with feeding in the order of rose flowers > 10% honey solution + bean pods > rose petals.

### Two-way ANOVA of 3 Digestive Enzyme Activities in *Frankliniella occidentalis* Based on Food Types and Generations

As shown in Table 5, the food types and generations showed a significant effect on the activities of 3 digestive enzymes (α-amylase, trypsin, and chymotrypsin) in second-instar larvae. And interaction between food types and generations significantly influenced the activities of 3 digestive enzymes. Similarly, the activities of 3 digestive enzymes were influenced significantly in adults by food types, generations, and interaction between them.

**Table 5.** Two-way ANOVA of digestive enzyme activities in *F. occidentalis* based on food types and generations

Source of variation	df	Second-instar larvae		Adults	
		*F*	*P*	*F*	*P*
α-Amylase					
Food types	2	557.401	**<0.001**	181.624	**<0.001**
Generations	4	108.425	**<0.001**	117.964	**<0.001**
Food types × generations	8	10.088	**<0.001**	12.312	**<0.001**
Trypsin					
Food types	2	222.742	**<0.001**	140.613	**<0.001**
Generations	4	79.502	**<0.001**	124.100	**<0.001**
Food types × generations	8	26.669	**<0.001**	32.206	**<0.001**
Chymotrypsin					
Food types	2	24.226	**<0.001**	107.912	**<0.001**
Generations	4	45.133	**<0.001**	33.943	**<0.001**
Food types × generations	8	10.866	**<0.001**	18.942	**<0.001**

Significant effects (*P* < 0.05) are indicated in bold. Summary results following generalized linear model ANOVA in SPSS Version 26.

### Effects of Food Types on α-Amylase Activity in WFTs of Different Generations

Changes in α-amylase activity in WFTs fed on different foods varied among generations (Table 6). After feeding on rose petals, a significant decrease in α-amylase activity in F_1_ second-instar larvae was observed, which increased to the levels observed in the control in the F_2_ generation. α-amylase activity increased steadily until the F_7_ generation but did not exhibit significant differences compared with that in the control. After feeding on rose flowers, α-amylase activity in second-instar larvae significantly increased with generations and was maintained at a higher level in the F_5_ generation. Changes in enzyme activity in second-instar larvae fed on 10% honey solution + bean pods were similar to those fed on rose flowers, and the enzyme activity was significantly higher in the F_3_ generation, which was maintained thereafter.

**Table 6.** Changes of α-amylase activity in *F. occidentalis* feeding on different foods for different generations

Food types	CK (kidney bean pods)	α-Amylase (mg/min/mg prot)				
		F_1_	F_2_	F_3_	F_5_	F_7_
Second instar larvae						
Rose petals	0.3525 ± 0.0079ab	0.2760 ± 0.0041cB	0.3195 ± 0.0036bC	0.3867 ± 0.0091aC	0.3777 ± 0.0126aB	0.3851 ± 0.0071aC
Rose flowers	0.3525 ± 0.0079d	0.4336 ± 0.0053cA	0.4887 ± 0.0103abA	0.4750 ± 0.0049bB	0.5164 ± 0.0029aA	0.5049 ± 0.0089aA
10% Honey solution + kidney bean pods	0.3525 ± 0.0079c	0.4118 ± 0.0102bA	0.4353 ± 0.0114bB	0.5407 ± 0.0017aA	0.5332 ± 0.0053aA	0.5384 ± 0.0057aB
Adults						
Rose petals	0.2751 ± 0.0026bc	0.2314 ± 0.0040dB	0.2645 ± 0.0012cB	0.3146 ± 0.0025aC	0.3038 ± 0.0042abB	0.3115 ± 0.0147aB
Rose flowers	0.2751 ± 0.0026d	0.2894 ± 0.0064cdA	0.3036 ± 0.0046cA	0.4111 ± 0.0032aA	0.3666 ± 0.0038bA	0.3512 ± 0.0018bA
10% Honey solution + kidney bean pods	0.2751 ± 0.0026d	0.3066 ± 0.0028cA	0.3226 ± 0.0081bcA	0.3419 ± 0.0042abB	0.3644 ± 0.0061aA	0.3452 ± 0.0081abB

Data in the table are mean ± SE. Different lowercase letters indicate significant differences in the same row, and different uppercase letters indicate significant differences in the same column in second-instar larvae or adults. The Tukey’s test was used to analyze significant differences at the *P* < 0.05 level.

Changes in α-amylase activity in second-instar larvae fed on the 3 food types varied in different generations. α-amylase activity in F_1_ and F_5_ second-instar larvae fed on rose petals was lower than that in those fed on other 2 food types, whereas α-amylase activity in the F_2_ and F_3_ second-instar larvae fed on rose flowers was higher than that in those fed on 10% honey solution + bean pods. In contrast, diverse patterns of α-amylase enzyme activity were observed in the F_3_ generation.

Changes in the α-amylase activity in adult thrips fed on the 3 food types were consistent with the increase in generations. The F_1_ adults fed on rose petals showed evidently decreased α-amylase activity, which increased from the F_2_ generation. Feeding of thrips with rose flowers resulted in increased α-amylase activity from the F_1_ generation, with a peak in the F_3_ generation, followed by a slight decrease. However, its activity remained higher than that in the control in the F_3_ and F_5_ generations. Feeding of thrips with 10% honey solution + bean pods caused an increase in α-amylase activity from the F_1_ generation compared with that in the control group, which peaked in the F_5_ generation.

Similar changes in α-amylase activity in adult thrips were observed in the F_1_, F_2_, and F_5_ generations after feeding on different food types, and the activity was lower when fed on rose petals. The highest α-amylase activity was observed in the F_3_ and F_5_ generations after feeding thrips with rose flowers. In contrast, the lowest activity was detected in thrips fed on rose petals in 5 generations (F_1_, F_2_, F_3_, F_5_, and F_7_), whereas no significant difference was observed between feeding with rose flowers and 10% honey solution + bean pods in the F_1_, F_2_, and F_5_ generations.

### Effects of Food Types on Trypsin Activity in WFTs of Different Generations

Trypsin activity in F_1_ second-instar larvae decreased significantly after feeding on rose petals (Table 7) and then increased to similar levels as the control in the F_2_ generation. The enzyme activity then continuously increased in the F_3_ generation but did not exhibit any significant difference compared with that in the control. Trypsin activity peaked in the F_5_ generation and maintained similar levels in the F_7_ generation. No significant change was observed in trypsin activity in F_1_ second-instar larvae after feeding on rose flowers. However, it increased and peaked in the F_2_ generation before decreasing in the F_3_ generation but showed higher levels than that in the control. In contrast, no significant differences in trypsin activity were observed among F_3_, F_5_, and F_7_ generations. After feeding on 10% honey solution + bean pods, trypsin activity in second-instar larvae of all generations was significantly higher than that in the control, with the highest level in the F_2_ generation; however, it was not significantly different from that in other generations.

**Table 7.** Changes of trypsin activity in *F. occidentalis* feeding on different foods for different generations

Food types	CK (kidney bean pods)	Trypsin (U/mg prot)				
		F_1_	F_2_	F_3_	F_5_	F_7_
Second instar larvae						
Rose petals	32.52 ± 1.15b	24.07 ± 1.33cB	32.13 ± 1.33bC	36.56 ± 0.96abB	41.83 ± 2.26aB	39.97 ± 1.72aC
Rose flowers	32.52 ± 1.15c	40.09 ± 1.15cA	81.55 ± 1.58aA	49.12 ± 3.21bA	53.68 ± 1.53bA	57.41 ± 1.00bA
10% Honey solution + kidney bean pods	32.52 ± 1.15c	44.85 ± 1.18bA	67.02 ± 1.64aB	47.86 ± 1.65bA	47.07 ± 1.46bA	48.37 ± 1.76bB
Adults						
Rose petals	19.70 ± 0.26c	17.14 ± 0.88cC	26.75 ± 1.65bC	33.39 ± 0.79aB	36.36 ± 0.92aAB	36.85 ± 1.20aB
Rose flowers	19.70 ± 0.26d	29.96 ± 0.72cA	60.68 ± 1.18aA	40.39 ± 0.52bA	40.35 ± 1.03bA	41.72 ± 0.40bA
10% Honey solution + kidney bean pods	19.70 ± 0.26c	24.58 ± 0.91cB	48.67 ± 2.42aB	33.70 ± 0.90bB	33.96 ± 1.55bB	32.60 ± 1.29bB

Data in the table are mean ± SE. Different lowercase letters indicate significant differences in the same row, and different uppercase letters indicate significant differences in the same column in second-instar larvae or adults. The Tukey’s test was used to analyze significant differences at the *P* < 0.05 level.

Feeding on rose petals resulted in the lowest trypsin activity in second-instar larvae in all generations. However, no significantly different changes in trypsin activities were observed between feeding on rose flowers and 10% honey solution + bean pods in different generations. No significant differences were detected in trypsin activities in F_1_, F_3_, and F_5_ second-instar larvae fed on rose flowers and 10% honey solution + bean pods. However, the enzyme activity in F_2_ second-instar larvae was higher than that in F_7_ second-instar larvae.

Adult thrips fed on rose petals and 10% honey solution + bean pods showed an evident increase in trypsin activity until the F_2_ generation. Similarly, the enzyme activity increased from the F_1_ generation in adults fed on rose flowers, whereas the enzyme activity in those fed on rose flowers and 10% honey solution + bean pods started to decrease after the F_3_ generation, but it showed significantly higher levels than that in the control.

Trypsin activity peaked in all generations after feeding adult thrips on rose flowers, but the activities differed in different generations when they were fed on rose petals and 10% honey solution + bean pods. Lower enzyme activities were detected in the F_1_ and F_2_ generations when thrips were fed on rose petals; however, the activities in thrips of the F_1_ and F_2_ generations were not significantly different from those of F_3_, F_5_, and F_7_ generations after feeding on 10% honey solution + bean pods.

### Effects of Food Types on Chymotrypsin Activity in WFTs of Different Generations

Changes in chymotrypsin activities in F_1_–F_3_ second-instar larvae fed on rose petals and 10% honey solution + bean pods were similar, and the activity was significantly higher than that in the control (Table 8). However, changes in chymotrypsin activities of F_5_ and F_7_ second-instar larvae were different between the 2 food types. Similarly, high chymotrypsin activity was observed after feeding on rose petals, but it decreased after feeding on 10% honey solution + bean pods. Thrips fed on rose flowers showed the highest chymotrypsin activity in the F_1_ generation, but it decreased with generations, with the lowest activity in thrips of the F_5_ generation.

**Table 8.** Changes of Chymotrypsin activity in *F. occidentalis* feeding on different foods for different generations

Food types	CK (kidney bean pods)	Chymotrypsin (U/mg prot)				
		F_1_	F_2_	F_3_	F_5_	F_7_
Second-instar larvae						
Rose petals	0.985 ± 0.108b	1.397 ± 0.038aB	1.345 ± 0.030aB	1.470 ± 0.021aB	1.302 ± 0.055aA	1.391 ± 0.028aA
Rose flowers	0.985 ± 0.108e	1.921 ± 0.078aA	1.824 ± 0.089abA	1.543 ± 0.025bcAB	1.185 ± 0.035dAB	1.489 ± 0.016cdA
10% Honey solution + kidney bean pods	0.985 ± 0.108b	1.594 ± 0.025aB	1.669 ± 0.084aB	1.624 ± 0.033aA	1.032 ± 0.069bB	1.233 ± 0.040bB
Adults						
Rose petals	0.773 ± 0.035b	1.029 ± 0.044abB	1.016 ± 0.079abC	1.269 ± 0.110aA	1.146 ± 0.035aA	1.203 ± 0.076aA
Rose flowers	0.773 ± 0.035d	1.416 ± 0.016bA	1.844 ± 0.045aA	1.419 ± 0.048bA	1.120 ± 0.031cA	1.175 ± 0.028cA
10% Honey solution + kidney bean pods	0.773 ± 0.035c	1.009 ± 0.032bB	1.402 ± 0.028aB	0.828 ± 0.020cB	0.735 ± 0.021cB	0.725 ± 0.009cB

Data in the table are mean ± SE. Different lowercase letters indicate significant differences in the same row, and different uppercase letters indicate significant differences in the same column in second-instar larvae or adults. The Tukey’s test was used to analyze significant differences at the *P* < 0.05 level.

The highest chymotrypsin activity was observed in F_1_ and F_2_ second-instar larvae fed on rose flowers, but no significant difference in enzyme activity was detected between the other 2 food types. The observed chymotrypsin activity in second-instar larvae until the F_3_ generation was higher feeding on 10% honey solution + bean pods than on rose petals. But the enzyme activity was diverse in the F_5_ and F_7_ generations.

Adult thrips fed on rose petals showed increased chymotrypsin activity in the F_1_ and F_2_ generations, which was not significantly different compared with that in the control. However, chymotrypsin activity increased significantly from the F_3_ generation. The highest chymotrypsin activity was observed in adults thrips fed on rose flowers in the F_2_ generation, which decreased with generations, and the lowest activity, which was still higher than that in the control, was detected in the F_5_ and F_7_ generations. Adult thrips fed on 10% honey solution + bean pods showed increased trypsin activity with generations and peaked in the F_2_ generation before decreasing to the control level in F_3_ generation.

Chymotrypsin activity in adult thrips fed on different foods varied with generations. In the F_1_ generation, chymotrypsin activity in adults fed on rose flowers was higher than that in those fed on rose petals and 10% honey solution + bean pods. In the F_2_ generation, chymotrypsin activity increased with feeding in the order of rose flowers > 10% honey solution + bean pods > rose petals. From the F_3_ to F_7_ generation, the enzyme activities in adult thrips fed on rose petals and rose flowers were higher than that in thrips fed on 10% honey solution + bean pods.

### Correlations Between Nutrient Contents and Digestive Enzyme Activities in WFTs

Tables 9 showed Pearson’s correlation coefficients between 3 nutrient contents (soluble sugar, protein, and lipid) and 3 digestive enzyme activities (α-amylase, trypsin, and chymotrypsin). There were obvious correlations among the contents of 3 nutrient related positively with the activities of α-amylase and trypsin in second-instar larvae and adults, respectively. But the chymotrypsin activity was not correlated significantly with the contents of soluble sugar and lipid in second-instar larvae and adults, respectively.

**Table 9.** Correlations between nutrient contents and digestive enzyme activities in *F. occidentalis*

Digestive enzymes	Nutrients		
	Soluble sugar	Protein	Lipid
Second-instar larvae			
α-Amylase	0.90*	0.88*	0.78*
Trypsin	0.67*	0.70*	0.73*
Chymotrypsin	−0.03	0.10	0.10
Adults			
α-Amylase	0.75*	0.80*	0.80*
Trypsin	0.53*	0.74*	0.61*
Chymotrypsin	−0.11	0.34*	0.18

*The data are Pearson’s correlation coefficients, and significance (*P* < 0.05). Summary results following Pearson’s correlation analyses in Origin version 2022.

## Discussion

The nutritional property of food types is a crucial factor that directly affects the performance and fitness of insects as well as their adaptation to different foods (Atakan and Uygur 2005, Wilson et al. 2019). Nutrient components, such as carbohydrates, proteins, and amino acids, are essential for insect growth and development (Friend 1958, Awmack and Leather 2002). Variation in the nutrition of food types can directly affect the nutrient uptake of insects, leading to changes in the physiological and biochemical properties of insects (Felton 1996, Wilson et al. 2019). In this study, changes in nutrient contents in 4 food types were evaluated, and the highest contents of soluble sugar (in 10% honey solution + kidney bean pods) and amino acid (in rose flowers) were found, respectively. The protein content of rose flowers was significantly higher than that of bean pods and rose petals, whereas its content was not significant compared with 10% honey solution + kidney bean pods. The changes in nutrient contents and digestive enzyme activities in thrips were also determined after feeding on different food types, and variations according to feeding time, generation, and insect stages were observed. This suggested that differences in the nutrient composition and contents of food types regulated the mechanisms of digestive metabolism in insects.

Energy obtained from food is critical for the growth, development, and reproduction of insects (Clissold and Simpson 2015, Bala et al. 2018). Thus, nutrient contents in insects depends on food resources, and its variation was used as an important indicator of feeding capacity and host adaptation (Brodbeck et al. 1993, Parra et al. 2012). A previous study showed that the host plant nutrient components, particularly proteins, free amino acids, and soluble sugars, could strongly affect the nutrient contents in insects, such as *Bradysia cellarum* and *B. impatiens* (Gou et al. 2020). Significant differences in protease and amylase activities were observed in *Helicoverpa armigera* larvae fed on host plant cultivars with varied total protein and carbohydrate contents (Hemati et al. 2012). In this study, the contents of different nutrients, such as soluble sugars, proteins, and amino acids, were different in the 4 tested food types. The thrips feeding on rose petals resulted in lower contents of soluble sugar, protein, and lipid in second-instar larvae and adults compared with those fed on other 2 food types. The highest sugar content was detected in thrips fed on 10% honey solution + kidney bean pods, whereas the highest protein and lipid content was observed in thrips fed on rose flowers. This could be explained by the higher soluble sugar and protein contents in rose flowers and 10% honey solution + kidney bean pods than in kidney bean pods. These results showed that the increase in nutrient content was associated with higher content of nutrients in WFTs. This was consistent with the results of a recent study, which reported that rose flowers at the full bloom stage exhibit significantly higher soluble sugar content than those at the bud stage (Chen et al. 2021). Among the food types tested in this study, bean pods coated with honey solution provided a more direct source of sugar for thrips than rose petals. Similarly, according to a previous study, the sugar content in adult bee worms fed on 10% honey solution was significantly higher than that of worms fed on other food types (Song and Luo, 2007). Considering that sugar content is a key factor controlling the occurrence of feeding hazards in insects, we speculated that sugar from rose flowers alters the levels of nutrients and biological enzymes in WFTs. A previous study showed that sucrose solution mixed food supplemented with rose pollen had a better feeding effect on WFTs (Shen et al. 2012). In addition, a study revealed that WFTs switched from gardenias with higher soluble sugar content to roses and gerbera with lower soluble sugars. The highest soluble sugar content in second-instar larvae and adults thrips was also observed after feeding on gardenias (Cao 2019).

The quality of foods or diets determines the growth, development, and reproduction of insects (Awmack and Leather 2002, Malinga et al. 2018). Higher amino acid and fatty acid content contributes to improve fecundity and lifespan of the dark-gill beetle *Holotrichia parallela* (Xu et al. 2019). Similarly, content ratio of proteins and carbohydrates in food significantly affected the growth, development, and reproduction of *Henosepilachna vigintioctopunctata* (Wang et al. 2018). Our previous analysis also showed that feeding thrips on rose flowers and 10% honey water + bean pods shortened their development duration, prolonged their life span, and enhanced their egg production. Thus, we speculated that the differences in insect life parameters were most likely associated with nutrient intake from foods.

Temporal changes in the physiological and biochemical levels of insects were observed after feeding on various food types. This study observed a decrease in the contents of soluble sugar and protein in both F_1_ second-instar larvae and adults thrips fed on rose petals, which increased after the F_2_ generation. Feeding of thrips on rose flowers and 10% honey solution + kidney bean pods increased the soluble sugar and protein contents in both second-instar larvae and adults from the F_1_ generation, suggesting that the lower nutrient levels in rose petals causes a decrease in the contents when feeding began; after 2 generations, thrips adapted to the foods and could meet their dietary requirement. In addition, rose flowers and 10% honey solution + kidney bean pods showed high contents of soluble sugar and protein, which increased from the F_1_ generation. The sugar and protein contents peaked in the F_2_ generation regardless of the food type, which not only indicated that thrips reached a stable level after 2 generations but also showed that nutrients need time accumulation after feeding. In contrast to the observed changes in soluble sugar contents, an increase in lipid content was observed from the F_1_ generation regardless of the food type, which indicated that the changes in lipid were not associated with the contents of foods. This might be due to the higher sugar content in pollen, nectar, and honey solution of flowers, enabling higher sugar intake by thrips after feeding on nutritious flowers (including pollen nectar) and bean pods coated with honey solution. The sugars can then synthesize large amounts of lipids through the glycolipid metabolic pathway in insects (Wei and Su 2016). During feeding on a more preferred food type, increased sugar intake from the food is stored. In contrast, to maintain normal body growth when feeding on a less preferred food type, insects will prolong the feeding time and consume more sugar.

Physiological enzyme activities are crucial for nutrient uptake from food for the normal growth, development, and reproduction of insects (Namin et al. 2014, Hemmati et al. 2022). The insect's digestive enzyme activity can reflect its capacity to use different food types (de Figueroa et al. 2011, Li 2015). Comparison of digestive enzyme activities in thrips fed on different food types in this study, showed higher activities after feeding with the rose flowers and 10% honey solution + kidney bean pods than the rose petals in most treatments regardless of the insect generation, which suggested that the rose petals was not a preferred food type for the thrips. Feeding on high nutrient source by the WFTs improved their digestive enzyme activities in order to cope with the rapid body development. Previous studies have revealed that the level of digestive enzyme activity indicated the utilization of nutrients and the degree of food addiction by insects (Wright et al. 2006, Namin et al. 2014, Meriño-Cabrera et al. 2018). Higher digestive enzyme activity after feeding on nutrient-rich hosts enables insects to rapidly decompose and digest a large amount of nutrients, ensuring their rapid growth and population increase. Jiang et al. (2017) demonstrated that under normal carbon dioxide concentration, the amylase activity of thrips feeding on host cucumber was significantly elevated than in the nonhost pepper, while trypsin activity after cucumber and bean plants feeding was significantly higher than that of chili peppers, indicating that enzyme activity is correlated with the host plant feeding. Borzoui et al. (2015) found a positive association between starch level in the diets and activity of amylase enzyme for their breakdown in *Trogoderma granarium*. The results were in agreement with other studies that reported higher digestive enzyme activity when insects fed on their preferred plants (Wright et al. 2006, Jiang et al. 2017). Similarly, Jiang et al. (2017) showed that activities of amylase and trypsin in adults *F. occidentalis* were significantly related to the host preference, which in contrast had lower activity when fed on a less preferred host.

In this study, the activities of α-amylase and trypsin in the second-instar larvae and adults, as well as trypsin activity in second-instar larvae decreased in F_1_ generation when thrips were fed on rose petals. Switching to foods with normal nutritional components requires time to adapt to the changes in the physical and chemical properties of food to ensure normal body development, which reduces both enzyme activity and rapid energy consumption. For example, *Helicoverpa armigera* regulated the digestive enzyme activity levels according to the composition of macromolecules in its food to obtain necessary nutrients and avoid the negative effects of nutritional imbalance (Kotkar et al. 2009, Simon et al. 2015). The α-amylase and trypsin activities in both the second-instar larvae and adults thrips increased from F_1_ generation after feeding on the rose flowers and 10% honey solution + kidney bean pods. This indicates that WFTs switched from foods with normal nutritional components to those with rich nutrients, leading to enhanced physiological enzyme activity to obtain more energy substances. The enzyme activities increased from the F_2_ generation regardless of which food was fed to the thrips, which indicated that the thrips had adapted to feeding on higher quality food after the first generation. Consistently, transferring two-spotted spider mites to new hosts decreased their fitness more than they rapidly adapted to the new host, before increasing their fitness within 5 generations (Miyazaki et al. 2015). Additionally, after 3 or more generations of adaptation, the digestive enzyme activities in the second-instar larvae and adults of WFTs became stable on transformed hosts, and similar enzyme activities were also reported in *Bemisia tabaci* (Li 2009, Zhou et al. 2010).

The chymotrypsin activity in the second-instar larvae fed on rose petals significantly increased and peaked in the F_1_ generation, which was contrary to the changes in α-amylase and trypsin activities, suggesting that WFTs needed to use higher chymotrypsin levels to obtain more proteins by hydrolysis. Behmer (2009) also demonstrated that insects maintained or boosted the levels of enzymes for deficient nutrients. Similar findings also showed that tryptase activities were significantly increased after transfer to nonpreferred broad bean plant in the F_1_ generation (Zhi et al. 2021). Notably, changes in digestive enzyme activities between the larval and adult stages were similar in most situations but were not the same. This might be because adult WFTs need to absorb nutrients for development and fecundity.

This study demonstrated that the differences in total nutrient contents in the 3 food types were likely responsible for significant differences in the nutrient and physiological activities in the larvae and adults thrips. In most treatments, higher sugar and protein contents as well as higher nutrient contents and α-amylase and trypsin activities were detected in rose flowers and 10% honey solution + kidney bean pods. However, a similar pattern was not observed for changes in lipid content and chymotrypsin activity in some generations. The physiological differences in thrips and the complex relationship between insects and host plant are likely affected by several factors. In addition, various digestive enzymes are present in insects, which coordinate with each other to ensure their normal growth, development, and reproduction.

The plant nutrients that are absorbed by insects can simultaneously carry plant secondary compounds with adverse effects to the health of the feeding insects, and can significantly affect their growth, development, and physiological enzyme activities (Cao et al. 2015, Li et al. 2018). Differences in nutrient content or ingestion of protease inhibitors can directly affect the level of digestive enzyme metabolism of insects (Guerreiro et al. 2021, Zamani Fard et al. 2022). Significant differences in the activities of amylase, protease, and lipase were observed in insects after feeding on different foods (Wang et al. 2018, Kotkar et al. 2009), which affected the ability of insects to digest and absorb exogenous energy substances. The change of enzyme activity contributed to the utilization and adaptability of insects to different foods (Zhao et al. 2015). Moreover, evaluation of insect adaptations to hosts should not only consider food digestion by insects, but also the detoxification metabolism. For example, under host variation, *B. tabaci* regulated the activity levels of detoxifying and digestive enzymes to cope with the nutritional values in different hosts (Deng et al. 2013).

Overall, this study assessed the changes in the contents of nutrients, such as soluble sugar, lipid, and protein and elucidated the nutritional level and digestive enzyme activity in the WFTs by switching feeding sources. When WFTs fed on foods rich in nutrients, the nutrition content and the digestive enzyme activities increased and attained stable levels after adapting to new foods. However, the molecular mechanisms of nutrient signal regulation and digestion in thrips still need to be further explored.
